# The Potential of Vitamin K as a Regulatory Factor of Bone Metabolism—A Review

**DOI:** 10.3390/nu15234935

**Published:** 2023-11-28

**Authors:** Huakai Wang, Yongxi Ma

**Affiliations:** 1 Department of Animal Nutrition and Feed Science, State Key Laboratory of Animal Nutrition, College of Animal Science and Technology, China Agricultural University, Beijing 100193, China; huakaiwhk@cau.edu.cn; 2 Institute of Animal Husbandry and Veterinary Medicine, Anhui Academy of Agricultural Sciences, Nongkenan Road No. 40, Hefei 230031, China

**Keywords:** bioavailability, bone health, dietary intake, dietary source, vitamin K, vitamin K-dependent protein

## Abstract

Vitamin K (VK), a fat-soluble vitamin, is essential for the clotting of blood because of its role in the production of clotting factors in the liver. Moreover, researchers continue to explore the role of VK as an emerging novel bioactive molecule with the potential function of improving bone health. This review focuses on the effects of VK on bone health and related mechanisms, covering VK research history, homologous analogs, dietary sources, bioavailability, recommended intake, and deficiency. The information summarized here could contribute to the basic and clinical research on VK as a natural dietary additive and drug candidate for bone health. Future research is needed to extend the dietary VK database and explore the pharmacological safety of VK and factors affecting VK bioavailability to provide more support for the bone health benefits of VK through more clinical trials.

## 1. Introduction

**Figure 1:**
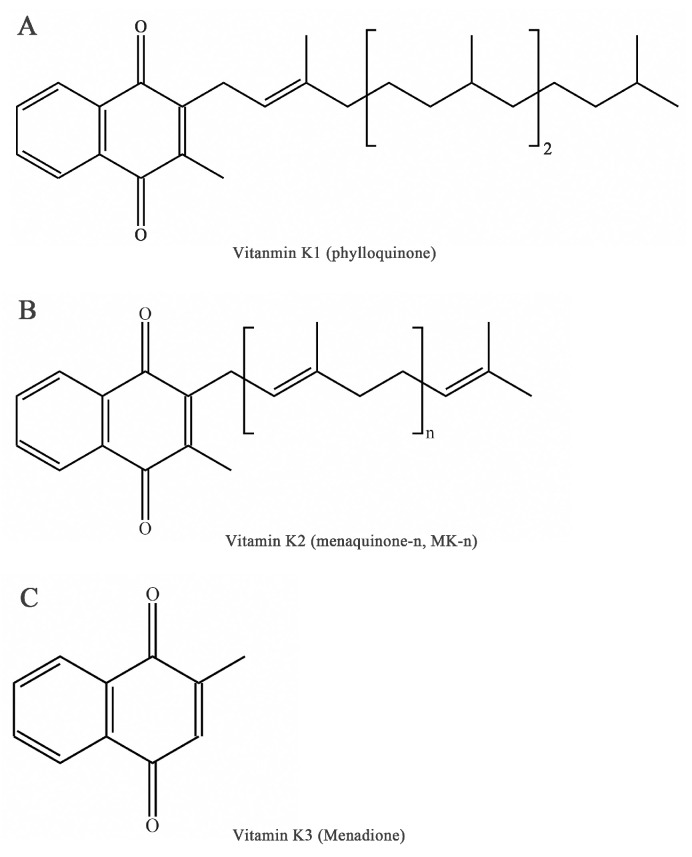
Chemical forms of vitamin K. (**A**) Chemical form of phylloquinone. (**B**) Chemical form of menaquinone. (**C**) Chemical form of menadione.

Vitamin K (VK), an essential lipid-soluble vitamin, is a generic term for a series of structurally related compounds of 2-methyl-1,4-naphthoquinone with a coagulation function [1]. The forms of VK differ in saturation and lengths of aliphatic side chains attached to the 3-position (Figure 1). Beyond the well-known biological function of coagulation [2], VK exerts a range of biological activities including antioxidation [3], anti-inflammation [4], and the prevention of cancer and cardiovascular disease [5,6,7]. Furthermore, emerging evidence of VK in bone metabolism suggests novel roles for VK in bone health [8].

In this review, we focus on the properties and mechanisms of action of VK on bone metabolism. First, we describe the history and nomenclature of VK, followed by its sources. Next, we summarize the bioavailability of VK, focusing on absorption, metabolism, and excretion. Then, the recommended intake and deficiency are presented. Finally, we discuss the beneficial effects and potential mechanisms of action of VK on bone metabolism. Therefore, we provide novel insights into the development of anti-osteoporosis drug candidates.

## 2. History and Nomenclature

The history of research on VK can be traced back to the work by Henrik Dam in 1929, in which it was found that the ingestion of foods extracted with nonpolar solvents in chicks resulted in a clotting defect [9]. The term “vitamin K” was first used by Henrik Dam in 1935. Subsequently, VK1 was characterized as 2-methyl-3-phytyl-1,4-naphthoquinone and synthesized by MacCorquodale et al. [10] in St. Louis, which was confirmed by Karrer et al. [11], Almquist and Klose [12], and Fieser [13].

It is worth noting that the current nomenclature in general used is the IUPAC–IUB Subcommittee Report on Nomenclature of Quinones. VK is used as a generic descriptor of a family of fat-soluble compounds that share a common 2-methyl-1,4-naphthoquinone ring. The three main forms are VK1 or phylloquinone, VK2 or menaquinone-n (MK-n), and VK3 or menadione (Figure 1). These derivatives can be differentiated by the 3-position: phylloquinone has an isoprenoid side chain, VK2 possesses a phytyl side chain and is characterized by 2–14 connected isoprenoid units (MK-2 to MK-14), and menadione has no side chain and is a synthetic analog [14].

## 3. Metabolism and Function

**Figure 2:**
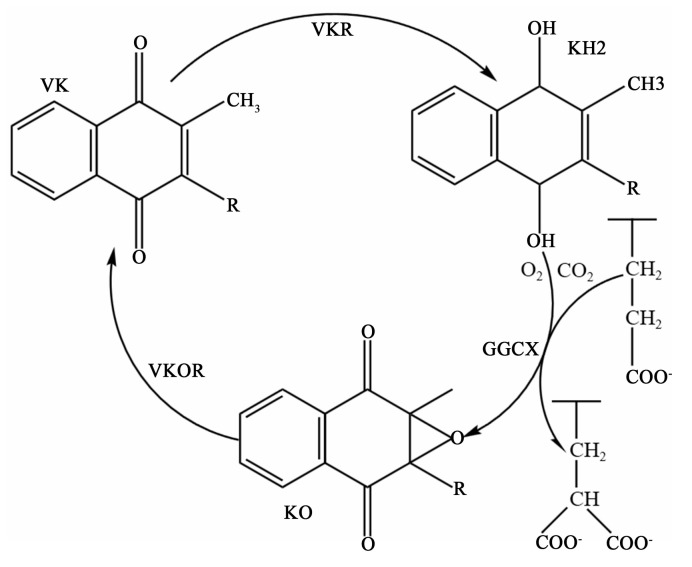
Vitamin K cycle. VK, vitamin K; KH2, a reduced form of vitamin K; KO, vitamin K 2,3-epoxide; GGCX, γ-glutamate carboxylase; VKOR, vitamin K epoxide reductase; VKR, vitamin K reductase.

VK metabolism is closely related to the activity of a microsomal carboxylase and is a cofactor of the enzyme. This enzyme relies on VK to convert the amino-terminal glutamate (Glu) residues of the precursor of VK-dependent proteins (VKDPs) to γ-carboxyl glutamate residues, after which the VKDPs can perform their normal physiological functions. The enzymes involved in this process include γ-glutamate carboxylase (GGCX), VK epoxide reductase (VKOR), and an as-yet-unidentified VK reductase (VKR). A reduced form of VK (KH2), CO2, and O2 are the cofactors of GGCX for catalyzing the carboxylation reaction. Concomitant with each carboxylation, KH2 is oxidized to VK 2,3-epoxide (KO). KO is converted back to KH2 through a two-step reduction using the VKOR and VKR in a pathway known as the VK cycle (Figure 2) [15]. At present, 17 types of VKDPs have been identified [16], including prothrombin; factors VII, IX, and X; anticoagulant proteins C, S, and Z; matrix Gla-protein (MGP); OC; growth-arrest sequence 6 protein (Gas6); Gla-rich protein (GRP); periostin; periostin-like factor; and four transmembrane Gla-proteins. Among them, the role of OC and MGP in bone health has been sufficiently detailed, while the role of other bone VKDPs seems less clear.

## 4. Various Sources

VK1 is a single compound present in photosynthetic organisms like green plants or vegetables [17,18] and constitutes approximately 75–90% of dietary sources of VK [19]. The content of VK in dietary sources is summarized in Table 1. Generally, all edible green plants can be considered an impartment source of VK1, among which kale and spinach are the richest vegetable sources of VK1, and their VK1 content can reach 817 μg/100 g and 387 μg/100 g, respectively [19]. Additionally, certain vegetable oils are also dietary sources of VK1, with soybean oil, rapeseed oil, and olive oil containing an average of 173, 123, and 53.7 μg/100 g of VK1, respectively [19,20]. As the predominant form of VK, VK1 is used in food supplements and drugs indicated in VK deficiency. As the natural vitamin requirement increases, the use of microalgae and cyanobacteria grown in bioreactors under highly controlled conditions to produce a significant amount of VK1 may be a promising option [18].

VK2 is present in natto, meat, and cheese and biosynthesized by certain obligate and facultative anaerobic bacteria. The form of VK2 produced depends on the strain of bacteria, while MK-4 is not produced by bacteria, but is formed from VK1 in humans and animals. UbiA prenyltransferase domain-containing protein-1 (UBIAD1) can cleave the side chain of VK1 to release VK3 and then prenylate it with geranylgeranyl pyrophosphate to produce MK-4 [24]. It is possible that both the release of VK3 in the GIT, with subsequent metabolism to MK-4 by UBIAD1, and the direct conversion of VK1 to MK-4 by UBIAD1 could be occurring [25,26]. According to Koivu-Tikkanen et al. [22] and Schurgers and Vermeer [19], MK-4 is the major VK2 in chicken (0.85–60 μg/100 g) or goose (31–369 μg/100 g) and typically accounts for 2.5% of dietary VK intake [27]. Besides meat, cheese and fermented vegetables also contribute to VK2 dietary consumption. Natto has been reported to contain a high concentration of MK-7 (998 μg/100 g), which accounts for 87.69% of total VK2 [19]. In addition, cheese also makes a substantial contribution to VK2 intake in the human diet; actual VK2 content depends on the type of cheese, the time of ripening, and the fat content [21,23]. Additionally, VK2 is also synthesized by the gut microbiota. MK-6, MK-7, and MK-8 are synthesized by Eubacterium lentum, Veillonella, and Escherichia coli, while MK-10 and MK-11 are synthesized by Bacteroides species [28,29]. However, the difference in fecal VK2 content was determined by a few genera within the gut microbiota [30], suggesting that VK2 content may be altered by diet-mediated alterations in gut microbiota composition.

## 5. Bioavailability

Previous studies and clinical reports have suggested the effect of VK on bone health, but there were still controversies. Thus, evidence of the absorption and metabolism of VK is necessary to evaluate the VK-induced effect on bone health.

### 5.1. Absorption

**Figure 3:**
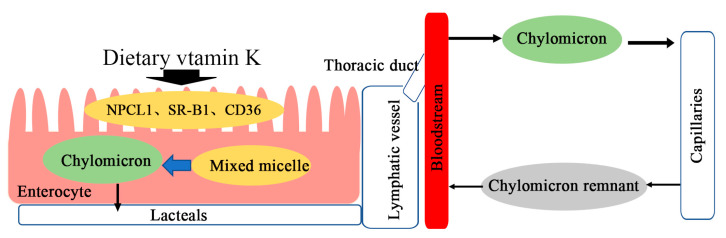
Intestinal absorption of dietary vitamin K.

The intestinal absorption of dietary VK is the same as that of other dietary lipids. After the dietary VK reaches the small intestinal lumen, it is incorporated into a mixed micelle of bile salts, the products of pancreatic lipolysis, and other dietary lipids [31]. Mixed micelles are absorbed by intestinal enterocytes and incorporated into chylomicrons (CMs). Subsequently, CMs are secreted from intestinal villi by exocytosis into the lymphatic capillaries (lacteals) and then join larger lymphatic vessels where they are released through the thoracic duct into the bloodstream [30,31]. In the bloodstream, CMs enter the capillary layer of peripheral tissues where they lose much of their triglyceride (TG) cargo through the action of lipoprotein lipase (LPL). Only a small fraction of the resulting chylomicron residue (CR) and central lipid core re-enter the circulatory system (31) (Figure 3). Niemann–Pick C1-like 1 (NPC1L1), scavenger receptor class B type I (SR-BI), and cluster of differentiation 36 (CD36) have been proposed to be involved in intestinal VK absorption [32]. Among them, NPC1L1 is a physiological importer of VK1 in the small intestinal, and SR-B1 and CD36 have VK1 uptake activity [33,34]. VK3, a synthetic VK, is widely used as a source of dietary VK for livestock, poultry, and laboratory animals, and it can be directly absorbed by a passive process from both the small intestine and the colon [35]. Subsequently, VK3 can be alkylated to MK-4 by UBIAD1 to exert biological activity.

There were few reports of the bioavailability of VK from different food sources. According to Gijsbers et al. [36], 1 h area under the curve (AUC) values for plasma VK1 from spinach with butter or without butter were 13.36% or 4.14% of that from a Konakion tablet. In a similar study, mean 9 h AUC values for plasma VK1 from fresh or cooked broccoli, fresh spinach, and romaine ranged from 9.04 to 22.87% of that from a Konakion tablet but did not significantly differ from each other [37]. This showed that the food matrix inhibits absorption of VK, as plasma response after ingestion of VK from plant-based foods is lower than that from purified VK [19]. Also, dietary fat contributes to the absorption of VK. Novotny et al. [38] reported that the bioavailability of VK1 from kale was 1–14%. Furthermore, Garber et al. [37] found that cooked broccoli had an earlier (2.7 h vs. 5.5 h) plasma concentration peak of VK1 and a higher (6.30 nmol/L vs. 3.97 nmol/L) bioavailability of VK1 than fresh broccoli, which indicated that meal components ingested with VK1 could significantly impact bioavailability. Moreover, a comparative study of the plasma time curves of synthetic VK1, MK-4, and MK-9 showed that the peak concentrations for VK1, MK-4, and MK-9 were reached at 4, 2.5, and 5 h postprandially [39], which suggested that dietary VK absorption might also be affected by its chemical structure.

### 5.2. Metabolism

Early studies have shown that VK is absorbed via the lymphatic system and then enters the bloodstream associated with CMs [40,41,42]. Notably, various malabsorption syndromes or biliary insufficiency reduced the absorption of VK. TG-rich lipoproteins containing very-low-density lipoproteins (VLDLs) and CMs predominantly transport VK1, while high-density lipoproteins (HDLs) transport MK-4 primarily in the postprandial state [39,42]. A comparative study of the physiological activities of VK2 homologs with different numbers of isoprene units in rats with hypoprothrombinaemia showed that MK-4 to MK-6 may be the most effective VK2 forms in nature [43]. Moreover, MK-7 to MK-13, synthesized by gut bacteria, were not effectively absorbed, and even if absorbed, they were less effective in improving VK deficiency [43].

The distribution of VK in the body is tissue-specific. According to Thijssen and Drittij-Reijnders [44], VK1 was recovered in all tissues with relatively high levels in the liver, heart, and pancreas, while MK-4 was recovered in most tissues with relatively high levels in the brain, kidney, and pancreas. In addition, there were sex-specific differences in tissue concentrations of VK1, MK4, some long-chain MKs, and dietary VK1 concentrations in C57BL6 mice [45].

Much less is known about the general mechanisms of tissue uptake of dietary VK, but they are in common with the general principles of fat absorption [31]. Niemeier et al. [44] reported that high levels of the CR receptors low-density lipoprotein receptor-related protein 1 (LRP1) and low-density lipoprotein receptor (LDLR) and lower levels of the very-low-density lipoprotein receptor (VLDLR) were expressed in human osteoblasts. Meanwhile, they found that LRP1 mediated VK uptake through CR endocytosis in osteoblasts, which was enhanced by the exogenous addition of apolipoprotein E (apoE) and lipoprotein lipase [46]. In common with osteoblasts, the uptake of VK by liver cells was verified to involve VLDLR-mediated CR endocytosis through apoE [31]. It is supposed that the uptake mechanism of VK accounts for the rapid removal of VK from the blood circulation.

Compared with VA, VD, and VE, VK has a lower concentration of circulation and tissue stores [27], indicating that the absorption of VK is intensively metabolized. Moreover, evidence from early isotopic work in humans showed that about 60–70% of dietary VK was ultimately excreted as catabolic products [42]. The process of the intermediary metabolism of VK, including catabolism and excretion, was much less understood. Previous studies have shown that unutilized VK3 was excreted as both glucuronide and sulfate conjugates of the quinol form in rats and rabbits [47,48,49]. In humans and animals, VK1 and VK2 share two common metabolites: 2-methyl-3-(3′-3′-carboxymethylpropyl)-1,4-naphthoquinone (5C-aglycone) and 2-methyl-3-(5-carboxy-3′-methyl-2′-pentenyl)-1,4-naphthoquinone (7C-aglycone) [50]. These two metabolites were excreted as water-soluble conjugates with glucuronic acid in the bile and urine primarily [50]. Thus, the measurement of urinary excretion can partly reflect the body’s VK status.

## 6. Recommended Intake and Deficiency

The present dietary reference values (DRVs) for VK are exclusively based on VK1. The recommended daily intake (RDI) or adequate intake of VK is mainly based on its function of coagulation. The adequate intake values of VK1 are 120 μg/d for adult men and 90 μg/d for adult women as recommended by the National Academy of Medicine in the US [51]. The World Health Organization and the Food and Agriculture Organization [52] recommend the RDI or adequate intake values for VK at 1 μg/d/kg body weight. Furthermore, the European Commission [52] recommended the RDI or adequate intake values for VK at 75 μg/d.

Nevertheless, studies have shown that VK requirements for maintaining bone health may be higher than the DRV [53,54,55]. Sokoll et al. [53] reported that VK intake with 100–420 μg/d could improve the γ-carboxylation status of osteocalcin (OC). According to Binkley et al. [56], VK intake with 1000 μg/d could maximally improve γ-carboxylate circulating OC. Moreover, an intervention study suggested that MK-4 supplementation at 1.5 mg/d improved bone quality in postmenopausal Japanese women without any substantial adverse effects [57]. Tsugawa et al. [54] demonstrated that the requirements of VK intake for bone health and normal blood coagulation were 155–188 μg/d and 54–62 μg/d by analyzing 1183 healthy adolescents. These studies indicated that VK deficiency has a more pronounced effect on bone than on blood coagulation. In addition, plasma VK levels also could not fully reflect the body’s VK status due to low circulating concentration, diet interference, or chronic disease [58,59]. VK deficiency prevents the adequate γ-carboxylation status of VKDPs, and therefore, undercarboxylated Gla-proteins have been used to detect subclinical vitamin K deficiency. Furthermore, a safe upper limit for dietary VK has not been established due to the deficiency of toxicity data.

## 7. Beneficial Properties for Bone Health

### 7.1. Vitamin K-Dependent Proteins

OC is the most abundant non-collagen protein in bone tissue, mainly secreted by osteoblasts and odontoblasts, and with a smaller amount produced by chondrocytes [60]. The undercarboxylated OC (ucOC) is an inactive form, while the active carboxylated form of OC has an increased affinity with free calcium ions and hydroxyapatite (HA), which subsequently inhibits the abnormal HA crystallization formation and cartilage mineralization speed and maintains the normal mineralization rate [61]. A previous study has shown that serum levels of ucOC may be more sensitive to VK status and can thus be used to detect subclinical VK deficiency [62]. Double-blind, randomized controlled trials reported that supplementation with ≥100 μg MK-7 in diets could improve OC γ-carboxylation status [63]. Moreover, long-term intake of this level of MK-7 contributed to maintaining γ-carboxylation status and improving bone health [63]. Earlier, Ma et al. [64] suggested that higher levels of serum ucOC not only reflected some degree of VK deficiency but also increased the risk of fracture. Wang et al. [8] reported that the addition of MK-4 could decrease serum ucOC levels and improve bone health in ovariectomized mice. Furthermore, in an in vitro study, it was found that OC may inhibit the early differentiation of osteoclasts mediated by Gprc6a [65]. However, Lambert et al. [66] suggested that osteocalcin-null rats presented higher trabecular bone volume and density and bone strength. According to results from clinical trials and animal studies, lower serum ucOC levels support the association between VK intake and bone health. The negative effect of OC on bone metabolism might be due to decarboxylation leading to the release of OC-bound calcium ions. Thus, additional well-controlled clinical studies are needed to clarify such mechanisms.

MGP is secreted by vascular smooth muscle cells, fibroblasts, and endothelial cells and exists in tissues, such as the heart, kidney, lung, skin, and arterial wall [67]. There are many forms of MGP, which differ in their phosphorylation and/or carboxylation states. Carboxylated MGP is a potent endogenous inhibitor of vascular calcification, which can prevent calcium phosphate precipitation by binding with bone morphogenetic protein 2 (BMP2) in blood vessels [68]. Marulanda et al. [69] demonstrated that overexpression of MGP in the vascular smooth muscle cells (VSMCs) could improve the low-bone-loss phenotype in Mgp-/- mice, which indicated that VK could increase the levels of carboxylated MGP to reduce vascular calcification and regulate calcium deposition into bone. Additionally, Evenepoel et al. [70] suggested that poor VK status could result in a higher level of dephosphorylated uncarboxylated MGP (dp-ucMGP) and induce inflammation, thereby reducing bone mineral density (BMD). Experimental in vitro data revealed that MGP treatment increased the mRNA and protein expression levels of Wnt3a, β-catenin, and Runx2, improving Wnt/β-catenin pathway-induced osteoblast proliferation, differentiation, and osteogenesis in MG63 cells [71]. Another study suggested that VK could upregulate the expression of MGP in primary osteoblasts and ovariectomized rats [72]. Evidence from mouse bone marrow macrophages (BMMs) indicated that the underlying suppressive mechanism of MGP on osteoclasts was associated with the modulation of the intracellular calcium flux and Src/Rac1 signaling [72]. Therefore, VK may serve as an important agent that contributes to the improvement of bone health through MGP.

### 7.2. Prevention and/or Treatment of Osteoporosis

Osteoporosis is a kind of systematic disorder closely related to lower bone strength and a higher risk of bone fracture. The incidence of osteoporotic fracture is rapidly increasing worldwide, and the number is expected to reach >300 million in 2040 with the continued increase in the aging population [73]. With the continued research on nutrition and clinical studies on dietary VK, evidence highlights its role in improving bone metabolism. The surveys and/or clinical trials on the effects of VK on bone health in humans over the past decade are summarized in Table 2 and Table 3.

An earlier cross-sectional analysis (in a population of community-dwelling elderly Japanese men) by Fujita et al. [74] showed that natto (containing 20 μg/pack VK1 and 380 μg/pack VK2) intake was higher in subjects with a lower level of serum ucOC, higher BMD, and lower risk of low BMD in the hip and femoral neck. The effects of natto on BMD have been implicated in serum ucOC levels as the association between natto intake and BMD became insignificant after adjustment for ucOC level [74]. Similarly, Kodama et al. [81] found that VK2 supplementation could reduce the serum level of ucOC and improve BMD at the radius in adults with cerebral palsy and osteoporosis. Sim et al. [82] reported that consuming VK1-rich green leafy vegetables could reduce serum ucOC. In a clinical study consisting of four women (aged 30–34 years) with pregnancy-associated osteoporosis, the researchers found that supplementation with 45 mg VK2 daily could weaken or eliminate back pain [75]. Interestingly, the Korea National Health and Nutrition Examination Survey consisting of 2785 men and 4307 women (aged ≥ 19 years) suggested that dietary VK intake was only positively correlated with femur BMD in men after adjusting for bone-related factors, while it was positively correlated with BMD both in the femur and lumbar in women [78]. Knapen et al. [77] noted that 180 μg/d MK-7 supplements could decrease the age-related decline in bone mineral content (BMC) and BMD in the lumbar spine and femoral neck, but not in the total hip. Moreover, supplementation with MK-7 (375 μg/d) could prevent age-related deterioration of trabecular bone in the tibia in postmenopausal women [80]. Recently, Evenepoel et al. [70] and Moore et al. [83] also found that VK status is associated with BMD in patients with end-stage renal disease and bone health in the risk population. Li et al. [89] also reported that MK-7 status is significantly associated with osteoporosis and could be considered a predictable biomarker in the diagnosis of osteoporosis in postmenopausal women. In contrast, a prospective cohort study including 1605 men and 1339 women in Hong Kong showed that dietary VK intake (men: 263.3 μg/d vs. 241.2 μg/d, women: 244 μg/d vs. 238.8 μg/d) could not reduce hip or nonvertebral fracture risk [84]. These discrepancies may be due to the high dietary intake of VK, which may have limited the ability to analyze the association between VK intake and fracture risk. On the other hand, Shikano et al. [86] found that VK improved bone turnover markers, while it did not affect BMD and fracture after 1.5 years in patients with osteoporosis on bisphosphonate therapy. Other similar studies suggested that combined therapy with risedronate and VK2 could not prevent vertebral fracture incidence in postmenopausal osteoporosis [85,87]. Moore et al. [88] also found that the addition of VK1 to oral bisphosphonate with calcium and/or vitamin D treatment in osteoporosis patients has a modest effect on parameters of hip geometry. However, Suzuki et al. [76] found that combined therapy with alendronate and VK2 reduced serum ucOC and improved lumbar spine and femoral neck BMD in postmenopausal patients with rheumatoid arthritis. In addition, lower serum VD and VK1 concentrations had a higher risk of hip fracture in elderly men and women [79]. Recently, Bartstra et al. [90] demonstrated that VK treatment did not affect arterial calcification or BMD in patients with type 2 diabetes mellitus and cardiovascular disease. Accordingly, dietary VK supplementation could reduce serum ucOC and improve BMD, while more studies are required to discuss the combined effect of VK and other anti-osteoporosis drugs or the effect of VK in different pathological conditions.

### 7.3. Mechanisms of Action

As discussed above, surveys or clinical trials have shown the beneficial effects of VK on bone health. Numerous studies have suggested that VK directly acts on VKDPs including OC and MGP to increase bone mineral deposition and bone formation [61,63,64,69,70]. Moreover, the protective effects of VK can be achieved by targeting multiple signaling pathways to regulate related transcription factors and/or proteins.

The underlying molecular mechanisms involved in mediating the osteoblast differentiation of VK have been reported in recent studies. Akbulut et al. [91] found that MK-7 promotes osteoblast maturation, thereby increasing osteogenic differentiation. Fusaro et al. [92] showed that VK2 as a transcriptional regulator of bone-specific genes through steroid and xenobiotic receptor (SXR) promoted the expression of osteoblastic markers such as ALP, osteoprotegerin (OPG), osteopontin (OPN), and MGP. Ichikawa et al. [93] demonstrated that cell surface markers of B-lymphoid lineage cells were activated by VK2 through SXR, which may be involved in osteoblast and osteoclast formation. Moreover, Ichikawa et al. [94] found that MK-4 stimulated growth differentiation factor 15 (GDF15) and stanniocalcin 2 (STC2) through the phosphorylation of protein kinase A (PKA) that was independent of the GGCX and SXR pathways. Furthermore, VK2 stimulated autophagy to promote osteogenic differentiation and mineralization in MC3T3E1 cells [95]. Subsequently, Cui et al. [96] reported that MK-4 promoted osteogenic differentiation through the Wnt/β-catenin signaling pathway in periodontal ligament stem cells (PDLSCs). In addition, VK2 improved osteogenic differentiation through the Bcl-6/STAT axis and IL-6/JAK/STAT signaling pathway in C3H10 T1/2 Clone 8 cells [97]. With experimental evidence from mouse models and MC3T3-E1 cells, Cui et al. [98] found that MK-4 prevents zoledronic acid-induced by inhibiting osteoblast apoptosis through suppression of cellular metabolic stresses in a SIRT1-dependent manner.

On the other hand, VK can play an osteoprotective role by antagonizing osteoclasts. Kameda et al. [99] indicated that VK2 suppressed osteoclastic resorption function by targeting osteoclasts for undergoing apoptosis. Also, Koshihara et al. [100] suggested that VK2 promoted osteoblastogenesis in human bone marrow cells while regulating osteoclastogenesis through the expression of RANKL/ODF rather than the OPG/OCIF pathway. A recent similar study also found that VK promoted the osteoblast-to-osteocyte transition while decreasing the osteoclastogenic potential of human primary osteoblast-like cells [101]. In addition, VK2 inhibited osteoclastogenesis by downregulating receptor activator of NF-κB (RANK) ligand (RANKL)-induced NF-κB activation in a γ-carboxylation-independent manner and promoted osteoblastogenesis by alleviating the suppression by tumor necrosis factor-α (TNF-α) of SMAD signaling induced by either BMP2 or transforming growth factor-β (TGF-β) [102]. In high-fat-diet-induced obese mice, VK supplementation alleviated high-fat-diet-induced bone loss by modulating osteoblast and osteoclast activities [103]. Noteworthily, combined treatment with VK2 and 1,25(OH)2D3 increased the levels of bone formation transcription factors such as OC, runt-related transcription factor 2 (RUNX2), Dlx5, activating transcription factor 4 (ATF4), and osterix (OSX) in primary osteoblasts harvested from the iliac crests of C57BL/KsJ lean (+/+) and obese/diabetic (db/db) mice [104]. Moreover, experimental in vivo data revealed that MK-4 inhibited osteoclast differentiation; decreased the mRNA expression of nuclear factor of activated T cells c1 (NFATc1), osteoclast-associated receptor (OSCAR), and cathepsin K (CTSK); and inhibited bone loss in ovariectomized mice [105]. Overall, evidence indicated that modulation of pathways by VK improves bone health through targeting osteoblasts and osteoclasts.

## 8. Conclusions and Future Perspectives

Collectively, studies have shown that VK is a natural bone-health-beneficial compound, although there is a great deal of variation between individuals in its adequate requirement, utilization, metabolism, and excretion in clinical studies. Certain studies indicated that VK2 may be more effective than VK1, and dietary VK plays its roles via several possible mechanisms, depending on differences in chemical construction or the length of the side chain. The requirements of VK differ in a wide range of humans because of the impact of diet composition, age, gender, and some chronic diseases. Thus, this review may be useful for developing dietary supplement guidelines for VK and discovering potential therapeutic targets.

Future research is suggested to focus on the following: (1) investigating the tissue specificity of VK absorption from different dietary sources and extending and validating the VK absorption database in foods; (2) the pharmacological safety evaluation of different sources of VK to determine its maximum effective and safe doses in both animals and humans; (3) exploring the effects and mechanisms of VK combined with other anti-osteoporosis drugs and supporting applications of VK as a candidate agent to prevent and treat osteoporosis in more clinical trials.

## Figures and Tables

**Table 1:** Vitamin K content of dietary sources (μg/100 g or μg/100 mL).

Type of Food	VK1	MK-4	MK-5	MK-6	MK-7	MK-8	MK-9	MK-10	MK-11	References
Vegetables										
Kale	817	-	-	-	-	-	-	-	-	Schurgers and Vermeer (2000) [19]
Spinach	387	-	-	-	-	-	-	-	-	
Broccoli	156	-	-	-	-	-	-	-	-	
Green peas	36	-	-	-	-	-	-	-	-	
Sauerkraut	25.1	0.4	0.8	1.5	0.2	0.8	1.1	-	-	
Natto	34.7	-	7.5	13.8	998	84.1	-	-	-	
Sauerkraut	22.4	0.43	0.86	1.59	0.23	0.89	1.5	-	-	Vermeer et al. (2018) [21]
Natto	32.1	-	7.2	12.4	996.5	82.4	-	-	-	
Meat										
Pig liver	1.2	1.08	-	-	1.6	2.5	0.6	0.8	-	Koivu-Tikkanen, Ollilainen, and Piironen (2000) [22]
Bovine liver	5.8	0.68	-	0.944	2.56	1.38	0.98	1.4	-	
Beef meat, roast	0.67	2.8	0.12	-	0.117	0.4	-	-	-	
Pork meat, chop	-	3.1	-	-	0.12	-	-	-	-	
Chicken meat, leg and thigh	-	60	-	-	-	-	-	-	-	
Beef	0.6	1.1	-	-	-	-	-	-	-	Schurgers and Vermeer (2000) [19]
Chicken breast	-	8.9	-	-	-	-	-	-	-	
Chicken leg	-	8.5	-	-	-	-	-	-	-	
Pork steak	0.3	2.1	-	-	-	-	-	-	-	
Pork liver	0.2	0.3	-	-	-	-	-	-	-	
Minced meat	2.4	6.7	-	-	-	-	-	-	-	
Salami	2.3	9.0	-	-	-	-	-	-	-	
Luncheon meat	3.9	7.7	-	-	-	-	-	-	-	
Hare leg	4.8	0.1	-	-	-	-	-	-	-	
Deer back	2	0.7	-	-	-	-	-	-	-	
Goose leg	4.1	31	-	-	-	-	-	-	-	
Goose liver paste	10.9	369	-	-	-	-	-	-	-	
Duck breast	1.9	3.6	-	-	-	-	-	-	-	
Minced	1.09	7.61	-	-	-	-	-	-	-	Vermeer et al. (2018) [21]
Pork cutlet	-	1.05	-	-	-	-	-	-	-	
Beef (meat)	-	1.39	-	-	-	-	-	-	-	
Beef (liver)	2.29	0.24	-	-	-	-	-	-	-	
Pork (meat)	-	1.36	-	-	-	-	-	-	-	
Pork (liver)	-	0.28	-	-	-	-	-	-	-	
Chicken (meat)	-	10.1	-	-	-	-	-	-	-	
Deer (back)	2.43	0.88	-	-	-	-	-	-	-	
Fish										
Rainbow trout, cultivated	0.56	3.1	0.09	-	0.2	-	-	-	-	Koivu-Tikkanen, Ollilainen, and Piironen (2000) [22]
Pike-perch	0.13	0.19	0.049	0.052	0.49	-	-	-	-	
Baltic herring	1.15	0.207	-	-	-	-	-	-	-	
Prawn	0.1	-	-	-	-	-	-	-	-	Schurgers and Vermeer (2000) [19]
Mackerel	2.2	0.4	-	-	-	-	-	-	-	
Herring	0.1	-	-	-	-	-	-	-	-	
Plaice	-	0.2	-	-	-	-	-	-	-	
Eel	0.3	1.7	-	-	-	-	-	-	-	
Salmon	0.1	0.5	-	-	-	-	-	-	-	
Mackerel	0.51	0.62	-	-	-	-	-	-	-	Vermeer et al. (2018) [21]
Eel	1.3	63.1	-	-	-	-	-	-	-	
Plaice	-	0.38	-	-	-	-	-	-	-	
Prawns	-	0.19	-	-	-	-	-	-	-	
Salmon	0.13	0.57	-	-	-	-	-	-	-	
Herring	0.11	0.07	-	-	-	-	-	-	-	
Oils and margarines										
Margarine	93.2	-	-	-	-	-	-	-	-	Schurgers and Vermeer (2000) [19]
Butter	14.9	15	-	-	-	-	-	-	-	
Corn oil	2.9	-	-	-	-	-	-	-	-	
Sunflower oil	5.7	-	-	-	-	-	-	-	-	
Olive oil	53.7	-	-	-	-	-	-	-	-	
Olive	30									Shearer and Bolton-Smith (2000) [20]
Olive (extra virgin)	80									
Rapeseed	123									
Soybean	173									
Dairy products										
Soured whole milk	0.217	0.57	0.293	0.17	0.41	2.01	4.7	-	-	Koivu-Tikkanen, Ollilainen, and Piironen (2000) [22]
Yogurt, plain	0.21	0.36	0.101	-	-	-	-	-	-	
Cheese, Edam type	1.91	3.3	1.05	0.56	1.26	10.5	30	0.86	-	
Cheese, Emmental type	2.58	5.23	-	-	-	-	-	-	-	
Cheese, Emmental type	3	6.1	-	-	-	-	-	-	-	
Whole milk	0.5	0.8	0.1	-	-	-	-	-	-	Schurgers and Vermeer (2000) [19]
Buttermilk	-	0.2	0.1	0.1	0.1	0.6	1.4	-	-	
Whole yoghurt	0.4	0.6	0.1	-	-	0.2	-	-	-	
Whipping cream	5.1	5.4	-	-	-	-	-	-	-	
Chocolate	6.6	1.5	-	-	-	-	-	-	-	
Hard cheeses	10.4	4.7	1.5	0.8	1.3	16.9	51.1	-	-	
Soft cheeses	2.6	3.7	0.3	0.5	1.0	11.4	39.6	-	-	
Curd cheese	0.3	0.4	0.1	0.2	0.3	5.1	18.7	-	-	
Egg yolk	2.1	31.4	-	0.7	-	-	-	-	-	
Brie (French)	4.92	12.5	-	-	-	-	-	-	-	Vermeer et al. (2018) [21]
Boursin (French)	4.55	8.93	-	0.11	0.33	0.82	0.91	-	-	
Camembert (French)	2.5	7.95	1.34	1.01	3.24	15.1	39.5	-	-	
Roquefort (French)	6.56	13.1	0.64	0.48	1.16	5.09	17.6	-	-	
Münster (French)	2.06	10.2	0.45	0.46	8.37	41.2	19.4	-	-	
Cheddar (British)	2.16	5.12	-	0.38	1.88	3.64	12.5	-	-	
Stilton (British)	10	0.94	0.6	1.4	6.63	29.8	-	-	-	
Mozzarella (Italian)	1.5	5.31	0.16	-	-	-	0.75	-	-	
Parmesan (Italian)	2.06	-	-	0.05	0.1	0.15	-	-	-	
Gorgonzola (Italian)	1.73	11.1	-	0.17	3.07	0.24	0.25	0.51	-	
Pecorino (Italian)	5.56	9.37	-	-	-	-	-	-	-	
Emmenthal (Swiss)	2.41	8.95	2.15	-	-	-	-	-	32.2	
Gruyère (Swiss)	2.5	5.15	1.38	-	-	-	-	-	-	
Raclette (Swiss)	1.55	4.77	0.4	0.31	1.13	4.77	20.9	-	-	
Gamalost (Norwegian)	0.18	1.03	0.62	0.29	0.97	5.12	4.4	2.2	-	
Norvegia (Norwegian)	4.37	5.1	-	0.3	1.33	5.25	2.95	-	-	
Regulate yogurt (full-fat)	0.4	0.7	-	-	-	-	13.2	1.6	8.4	Fu et al. (2017) [23]
Regulate yogurt (fat-free)	-	-	-	-	-	-	-	-	-	
Greek yogurt (full-fat)	0.3	0.8	-	-	-	-	14.8	1.8	8.7	
Greek yogurt (fat-free)	-	-	-	-	-	-	-	-	-	

**Table 2:** Summary of the effects of vitamin K (VK) on bone health in humans over the past decade. (vitamin K has a beneficial effect on bone).

Study Population	Interventions	Results	Reference
Healthy men (≥65 years, n = 1662)	Natto, <1 pack/week vs. 1 pack/week vs. several packs/week vs. 1 pack/day and more; 4–5 years	High natto intake was associated with lower ucOC and higher BMD	Fujita et al. (2012) [74]
Women with multiple vertebral fractures related to their normal pregnancies (31.5 years, n = 4)	VK2 (45 mg/d); 5 months to 1 year	Their back pain had completely disappeared	Tsuchie et al. (2012) [75]
Postmenopausal rheumatoid arthritis patients with untreated osteoporosis or osteopenia (young adults, n = 62)	VK2 (45 mg/d) plus alendronate (35 mg/week) vs. alendronate (35 mg/week); 1 year	Combined therapy increases lumbar spine and femoral neck bone density	Suzuki et al. (2013) [76]
Healthy postmenopausal women (55 to 65 years, n = 244)	MK-7 (180 μg/d) vs. placebo; 3 years	Prevention of bone loss	Knapen et al. (2013) [77]
Adults (>19 years, n = 7092)	Total VK, men: ≤58.07 (μg/d) vs. 58.09–121.91 (μg/d) vs. ≥121.93 (μg/d); women: ≤58.23 (μg/d) vs. 58.32–129.42 (μg/d) vs. ≥129.45 (μg/d)	Low dietary VK intake was associated with low bone mineral density	Kim, Kim, and Sohn (2015) [78]
Men and women (65 to 75 years, n = 21,774)	Serum VK and VK concentrations, high VK and high VD vs. high VK and low VD vs. low VK and high VD vs. low VK and low VD	An increased risk of hip fractures in elderly men and women with low concentrations of both VD and VK1	Finnes et al. (2016) [79]
Postmenopausal women (60 to 80 years, n = 148)	MK-7 (375 μg/d) vs. placebo; 1 year	Protection of trabeculae	Rønn et al. (2016) [80]
Adults with cerebral palsy and osteoporosis (n = 16)	VK2 (45 mg/d); 1 year	A positive effect on bone mineral density	Kodama et al. (2017) [81]
Patients with end-stage renal disease (54.7 ± 12.7 years, n = 468)	Serum dp-ucMGP, 300–862 nmol/L vs. 864–1447 nmol/L vs. 1465–10717 nmol/L	Poor VK status is associated with low bone mineral density	Evenepoel et al. (2019) [70]
Men and women (40 to 74 years, n = 30)	Phase 1 VK1 (164.3 μg/d) vs. phase 2 VK1 (9.4 μg/d) vs. none; three 4-week experimental phases	Reduce serum total OC and ucOC	Sim et al. (2020) [82]
Women with postmenopausal osteoporosis (68.7 years, n = 374)	Prevalent fractures vs. no fractures	VK status is associated with bone health including fracture risk and bone strength	Moore et al. (2020) [83]

ucOC, uncarboxylated osteocalcin; BMD, bone mineral density; VD, vitamin D; dp-ucMGP, dephosphorylated uncarboxylated matrix Gla-protein.

**Table 3:** Summary of the effects of vitamin K (VK) on bone health in humans over the past decade. (vitamin K has no effect on bones).

Study Population	Interventions	Results	Reference
Healthy men and women (≥65 years, n = 2944)	VK1, men: 266.7 μg/d vs. 240.9 μg/d; women: 239.8 μg/d vs. 238.9 μg/d; 1 year	Hip or nonvertebral fracture risk was not associated with dietary VK intake	Chan, Leung, and Woo (2012) [84]
Women with postmenopausal osteoporosis (>60 years, n = 101)	Risedronate and VK2 vs. risedronate; 1 year	No difference in vertebral fracture incidence	Kasukawa et al. (2014) [85]
Patients with systemic autoimmune diseases (≥51.1 years, n = 60)	Concomitant administration of bisphosphonate in all patients, VK2 (45 mg/d) vs. none; 1.5 years	No difference in bone mineral density and fracture rate	Shikano et al. (2016) [86]
Women with osteoporosis (≥65 years, n = 1983)	VK2 (45 mg/d) plus risedronate (2.5 mg/d or 17.5 mg/week) vs. risedronate (2.5 mg/d or 17.5 mg/week); 2 years	Concurrent treatment with VK2 and risedronate has worse effect compared with monotherapy with risedronate in terms of fracture prevention	Tanaka et al. (2017) [87]
Patients with osteoporosis (68.7 years, n = 105)	VK1 (1 mg/d) vs. MK-4 (45 mg/d) vs. placebo; 18 months	No difference in parameters of hip geometry	Moore et al. (2023) [88]

## Data Availability

The data that support the findings of this study are available from the corresponding author upon reasonable request.
